# Supplementary material for: High SARS-CoV-2 seroprevalence in Karaganda, Kazakhstan before the launch of COVID-19 vaccination
Source: PLoS One. 2022 Jul 27;17(7):e0272008. doi: 10.1371/journal.pone.0272008 (PMC9328563; doi:10.1371/journal.pone.0272008)
Supplement: Attachment — Submitted filename: PONE_Kadyrova_Response to Reviewer Comments.pdf [file pone.0272008.s002.pdf]

**Reviewer #1: The paper is well written and is relevant to current situation. Coming from one of the "stans" myself I realize the level of underreporting for such data. This makes it easier for me to understand the situation and importance of this paper. However, I have several concerns:**

**Authors' response:** We are very appreciative of the reviewer's positive view of our work and thank the reviewer for the insightful comments and questions. Please kindly find our point-by-point responses to the reviewer's remarks below.

**1. The history and situation with under reporting in the region is not fully explained.**

**Authors' response:** Thank you for raising this point. To address this comment, we have now added additional information to the Introduction to describe the situation with regard to COVID-19 under-reporting in Kazakhstan (p3, lines 15-23 and p4, lines 1-3).

**2. The cohort chosen cannot be generalized to the whole population - this is mentioned in the paper, but I think it would be good to provide more details about reasons for choosing this particular cohort (availability, connections, etc)**

**Authors' response**: Thank you for this very valid comment. This cohort was chosen for the serologic studies owing to funding availability and perceived feasibility in the context of the readily available resources and active participant recruitment within the infrastructure of the larger clinical trial. We have now added this information to the Materials and Methods (p4, lines 15-18).

**3. Last, but not least, I felt that there is something missing in conclusion. Specifically, lack of recommendation for further studies and possible actions. It was established that the prevalence of COVID-19 in the region is much higher than officially reported. What can be recommended to the government, how to improve reporting in general, how this underreporting is connected to uptake of vaccinations? If possible this information can be added to the paper to make it more convenient complete.**

**Authors' response:** Thank you for this suggestion! We have modififed the Discussion/Conclusions (p 12, lines 9-23) to include our recommendations with regard to improving case reporting and pandemic surveillance using serological screening and alternative approaches.

**Reviewer #2: Dear Authors! On acquainting with the presented paper, I found no significant remarks in the Methodology, Results presentation, and the Discussion section. The topic's relevance cannot be considered overestimated, although the tension of the Covid-19 pandemic has significantly decreased. Given the chance of the possible upcoming pandemics, the global scientific community strives to acquire a deeper understanding of all the details of the present pandemic process. In this relation, information from the countries with a comparatively low level of healthcare, such as Kazakhstan (LMICs out of the EU), appears to be particularly valuable. A study on Sputnik V preceded this work, and linked preprints from the MedRxiv Yale were placed into the References. In the methodology narration, the solution to use the manufacturers' cutoff values as being more suitable was relevant, thus indicating the quality of the work done. The dubious moment was the number of pre-pandemic samples (N 10), though it seems that this circumstance did not interfere with the validity of the results obtained.**

**The only I would suggest is to change your title slightly by inserting the brackets to lessen the number of commas: "High seroprevalence of SARS-CoV-2 antibodies in Karaganda (Kazakhstan) before the launch of COVID-19 vaccination" I found this conclusion the most important: "...serologically assessed SARS-CoV-2 exposure in this cohort was 14-15-fold higher than the reported all-time national and regional COVID-19 prevalence; the unusually high SARS-CoV-2 seroprevalence exceeding the estimates for many other countries." These data allow understanding of the shortcomings and pitfalls in the preventive strategies against the pandemic held in Kazakhstan. For instance, the paper showed the extremely low effectiveness of the screening by currently applied means. Albeit all the screened samples were negative, 59.2% of them turned out to be positive for anti-S IgA. I highly appreciated the integrity in presenting your data.**

**Authors' response:** We thank the reviewer for a very thoughtful and positive review of our work!

We agree with the reviewer that the original title of the manuscript appeared somewhat cumbersome, therefore we have adjusted it to: “High SARS-CoV-2 seroprevalence in Karaganda, Kazakhstan before the launch of COVID-19 vaccination.” We would be happy to replace the comma with brackets and ultimately leave this decision to the reviewers' discretion.

**Editorial Comments:**

**- Briefly explain the purpose of the pre-pandemic group in the Methods as well;**

**Authors' response:** Done, please see p 5, lines 8-11.

**- Explain which tests were used for which variables (instead of 'as appropriate');**

**Authors' response:** Done, please see p 7, lines 13-18.

**- Please kindly provide the reference number of the ethical clearance for the larger study within which this serologic survey was performed;**

**Authors' response:** Done, we have added this information to the "Ethics Statement" (p 7, lines 19-22).

**- Results are given for 99 subjects in the Table and for 98 subjects in the Results, please either correct or explain this discrepancy;**

**Authors' response:** We apologize for the the lack of clarity here. Two out of 100 participants were excluded from S-IgA testing due to insufficient sample volume. However, because one of these two participants tested S-IgG+, they were still included in the "Prior COVID-19" category" despite the missing S-IgA result. We have now made edits to both the Table (see Table 1 footnote) and the Results (p.8, lines 8-16) to clarify this aspect.

**- It is not clear whether 55 (methods) or 35 (results) subjects were selected for the neutralization assay, and by which criterion;**

**Authors' response:** We are sorry for the confusion here. The neutralization assays were performed on 55 samples (of which 35 were classified as Prior COVID based on the S-IgG and S-IgA ELISA), as stated in the Methods. We have now clarified this in the Results (p8, lines 7-19).

**- Please only use percentages in the table, as the numbers can be inferred thanks to the total in the top row (you can write "Results are expressed as percentages unless specified otherwise" in the title of the table; consistently, specify that for continuous variables you are providing the mean (and SD?); finally, the tests used should be explained as a note, not within the title of the table.**

**Authors' response:** Thank you for these suggestions. We have edited the Table as requested by the Editor and among other edits specified in the Table footnote the following: "Continuous and categorical variables are provided as median/interquartile ranges and percentages, respectively."

**Journal Requirements:**

**When submitting your revision, we need you to address these additional requirements.1. Please ensure that your manuscript meets PLOS ONE's style requirements, including those for file naming. The PLOS ONE style templates can be found at**

**https://journals.plos.org/plosone/s/file?id=wjVg/PLOSOne_formatting_sample_main_body.pdf and**

**https://journals.plos.org/plosone/s/file?id=ba62/PLOSOne_formatting_sample_title_authors_affiliations.pdf**

**Authors' response:** Thank you for pointing this out. We have reformatted the manuscript in accordance with the journal requirements.

**2. We note that the grant information you provided in the 'Funding Information' and 'Financial Disclosure' sections do not match. When you resubmit, please ensure that you provide the correct grant numbers for the awards you received for your study in the 'Funding Information' section.**

**Authors' response:** Thank you, we have double checked this section and confirm that the funding information is correct.

**3. Thank you for stating the following in the Acknowledgments Section of your manuscript:**

**"The study was funded by the Ministry of Education and Science of the Republic of Kazakhstan (AP09259123) and, in part, by the Nazarbayev University grant #280720FD1902 to GH. SY was supported, in part, by a M.G. DeGroote Postdoctoral Fellowship."**

**We note that you have provided funding information that is not currently declared in your Funding Statement. However, funding information should not appear in the Acknowledgments section or other areas of your manuscript. We will only publish funding information present in the Funding Statement section of the online submission form.**

**Please remove any funding-related text from the manuscript and let us know how you would like to update your Funding Statement.**

**Authors' response:** Thank you- we have now removed this information from the manuscript.

**Currently, your Funding Statement reads as follows:**

**"The study was funded by the Ministry of Education and Science of the Republic of Kazakhstan (AP09259123, https://www.gov.kz/memleket/entities/edu?lang=en) to IK and, in part, by the Nazarbayev University (https://nu.edu.kz/) grant #280720FD1902 to GH. SY was supported, in part, by a M.G. DeGroote Postdoctoral Fellowship.The funder of the study had no role in study design, data collection, data analysis, data interpretation, or writing of the report. All authors had full access to all the data in the study and the lead authors (IK, SY, DB) had final responsibility for the decision to submit manuscript for publication."**

**Please include your amended statements within your cover letter; we will change the online submission form on your behalf.**

**Authors' response:** Thank you -we have provided the amended version of the funding statement in the Cover letter.
